# New *SLC22A12* (URAT1) Variant Associated with Renal Hypouricemia Identified by Whole-Exome Sequencing Analysis and Bioinformatics Predictions

**DOI:** 10.3390/genes14091823

**Published:** 2023-09-20

**Authors:** Ana Perdomo-Ramírez, Elena Ramos-Trujillo, Félix Claverie-Martín

**Affiliations:** 1 Unidad de Investigación, Hospital Universitario Nuestra Señora de Candelaria, 38010 Santa Cruz de Tenerife, Spain; atter_rad@hotmail.com (A.P.-R.); eramostr@ull.edu.es (E.R.-T.); 2 Departamento de Medicina Física y Farmacología, Facultad de Ciencias de la Salud, Sección Medicina, Universidad de La Laguna, 38071 Santa Cruz de Tenerife, Spain

**Keywords:** renal hypouricemia, glucose transporter 9, urate transport 1, *SLC2A9*, *SLC22A12*, rare disease, mutation, whole-exome sequence analysis

## Abstract

Renal hypouricemia (RHUC) is a rare hereditary disorder caused by loss-of-function mutations in the *SLC22A12* (RHUC type 1) or *SLC2A9* (RHUC type 2) genes, encoding urate transporters URAT1 and GLUT9, respectively, that reabsorb urate in the renal proximal tubule. The characteristics of this disorder are low serum urate levels, high renal fractional excretion of urate, and occasional severe complications such as nephrolithiasis and exercise-induced acute renal failure. In this study, we report two Spanish (Caucasian) siblings and a Pakistani boy with clinical characteristics compatible with RHUC. Whole-exome sequencing (WES) analysis identified two homozygous variants: a novel pathogenic *SLC22A12* variant, c.1523G>A; p.(S508N), in the two Caucasian siblings and a previously reported *SLC2A9* variant, c.646G>A; p.(G216R), in the Pakistani boy. Our findings suggest that these two mutations cause RHUC through loss of urate reabsorption and extend the *SLC22A12* mutation spectrum. In addition, this work further emphasizes the importance of WES analysis in clinical settings.

## 1. Introduction

Renal hypouricemia (RHUC) is a genetic disorder caused by defective renal reabsorption of uric acid (UA) in the proximal tubule (PT) [1,2,3]. RHUC patients present low serum levels of UA, high fractional excretion of UA (FE UA), and increased predisposition to exercise-induced acute renal failure, EIARF) and nephrolithiasis [4,5,6,7,8,9]. Two types of RHUC are distinguished based on the mutated gene; RHUC type 1 (OMIM # 220150) is caused by inactivating mutations in the *SLC22A12* gene that encodes urate transport 1 (URAT1), a member of the organic anion transporter family [4,10,11], while RHUC type 2 (OMIM # 612076) is the result of loss-of-function mutations in *SLC2A9* encoding glucose transporter 9 (GLUT9, a member of the GLUT family of hexose transporters) [6,12,13]. URAT1 and GLUT9 are UA transporters involved in the reabsorption of UA in the proximal renal tubule [14]. The URAT1 protein contains 12 predicted transmembrane domains (TMDs), a long cytoplasmic loop between TMDs 6 and 7, cytoplasmic amino and carboxy ends and localized to the apical membrane of the proximal tubule epithelial cells [10]. *SLC2A9* generates two GLUT9 isoforms by alternative pre-mRNA splicing, GLUT9L and GLUT9S, which differ only at their amino termini and contain 12 predicted transmembrane helices and cytoplasmic amino and carboxy termini [15,16,17]. In the kidney, GLUT9L is expressed at the basolateral membrane of the epithelial cells of the proximal tubule and is responsible for UA reabsorption, whereas GLUT9S is localized to the apical membrane of collecting duct cells [16,18]. Both the GLUT9 variants facilitate urate uptake [19]. It has been suggested that in the proximal tubule UA is reabsorbed from the lumen by URAT1 located at the apical membrane, while GLUT9 facilitates the basolateral exit of the reabsorbed UA to the peritubular interstitium and the blood [12].

Loss-of-function mutations in these two transporters impair UA reabsorption resulting in reduced serum levels of UA and increased UA levels in urine. Nevertheless, it has been reported that homozygous *SLC2A9* mutations cause a more severe hypouricemia than homozygous *SLC22A12* mutations [4,6,8,9,18,20]. This difference can be explained because the reabsorption of UA is mediated not only by URAT1 but also by other apical UA transporters like the organic anion transporters OAT4 and OAT10, whereas the UA release into to the blood is facilitated exclusively by the basolateral GLUT9L transporter [6]. Consequently, the loss of URAT1 function causes a partial defect in UA reabsorption, while the loss of GLUT9 activity prevents UA reabsorption by all the apical transporters producing a total reabsorption defect.

To date, more than 200 cases harboring mutations in the *SLC22A12* gene, and approximately 30 cases with mutations in *SLC2A9,* have been described worldwide. RHUC patients have been reported among diverse ethnic populations and across geographically distant countries. The first reported cases of RHUC were from Japan, where *SLC22A12* variants p.(W258X) and p.(R90H) are frequent causes of RHUC type 1 [21,22,23]. In this country, the prevalence of RHUC is estimated to be approximately 0.3% [13,24], but a large-scale epidemiological survey searching RHUC prevalence is absent [2]. These two mutations have also been detected in RHUC type 1 patients from South Korea [5]. RHUC cases have also been described in Caucasian, Roma, and Jewish populations from different countries including Macedonia, Czech Republic, Spain, Israel, and Pakistan [8,9,20,25,26]. More recently, several cases from China have been reported [27,28]. The main cause of RHUC type 1 in Roma populations of Czech Republic, Slovakia, and Spain are *SLC22A12* variants p.(L415_G417del) and p.(T467M), which are present in very high frequencies in these populations (5.6% and 1.9%, respectively) [8,25,26,29]. On the other hand, *SLC2A9* mutation p.(T125M) is the main cause of RHUC type 2 among Spanish patients of Caucasian origin [9].

In this study, we report the clinical data of two new RHUC cases and the identification of the respective causative mutations, one of which has not been previously identified.

## 2. Patients and Methods

### 2.1. Patients

#### 2.1.1. Case 1

The proband was an 11-year-old Spanish Caucasian boy who was born prematurely at 32 weeks after in vitro fertilization (triplet pregnancy). There was paternal consanguinity but no family history of kidney disease. He was being treated with methylphenidate for attention-deficit/hyperactivity disorder (ADHD). In a routine blood test, a serum UA value of 0.8 mg/dL was detected and confirmed. His fractional excretion of UA was 54% and his serum creatinine was normal (0.63 mg/dL). Hypercalciuria, glycosuria, and proteinuria were not detected. The estimated glomerular filtration, acid-base balance, and abdominal ultrasound were normal. A clinical diagnosis of renal hypouricemia was made. His twin sister had low serum levels of UA (0.7 mg/dL), high fractional excretion of urate (40%), and a serum creatinine of 0.52 mg/dL. Their mother had normal serum levels of urate. Given the suspicion of renal hypouricemia and after informed consent, a genetic study was performed on both siblings and their mother.

#### 2.1.2. Case 2

The patient was a 3-year-old boy of Pakistani origin born after an uneventful pregnancy and delivered at term. His parents reported that there is no parental consanguinity. In a routine analysis requested for language delay and suspicion of autism spectrum disorder (ASD), very low levels of uric acid in serum were found (<0.2 mg/dL), which were later confirmed. His fractional excretion of urate was high (FE UA 117%) and his serum creatinine was normal (0.37 mg/dL). Levels of glucose, urea, creatinine, calcium, sodium, potassium, and chloride were all within the normal range. The abdominal ultrasound was normal. A genetic study was performed on the patient and his parents to confirm the clinical diagnosis of renal hypouricemia.

### 2.2. Whole Exome Sequence Analysis and Variant Assessment

The Ethics Committee of the Hospital Universitario Nuestra Señora Candelaria (Santa Cruz de Tenerife, Spain) approved this study (protocol PI 56-17). After obtaining written informed consent from the patients’ parents in accordance with the Declaration of Helsinki, patients and parents underwent trio-whole exome sequencing. Peripheral blood (3 mL, in an EDTA tube) and urine samples were collected for biochemical and genetic analysis. The genomic DNA of the patients and their parents was extracted using the GenElute Blood Genomic DNA kit (Sigma-Aldrich, St. Louis, MO, USA) following the manufacturer’s instructions. DNA samples were sent to Macrogen Inc. (Seoul, Republic of Korea), where whole exome sequencing (WES) analysis was performed using an Illumina platform (Sureselect V6 + NovaSeq 6000 150 PE (150 × 2 bp) 18 Gb/sample (100× on target)). The mean value of the total % on target coverage obtained for the three samples was 63%. The affecting variants were identified using a gene panel of known genes associated with renal UA transport after excluding variants in introns and synonymous variants. Additionally, a second analysis of rare variants was performed after excluding variants in introns and UTRs, synonymous variants, variants in databases with frequency > 1%, variants with quality < 100, and variants with a low probability to be damaging according to SIFT, LRT, MutationTaster, PROVEAN, MutPred, and FATHMM. The variants identified using WES were confirmed by Sanger sequencing (Macrogen Inc., Madrid, Spain) and cosegregation analysis. We used FinchTV (Geospiza Inc., Denver, CO., USA) to read the .ab1 files. Several databases of genetic variants including the Genome Aggregation Database (gnomAD, https://gnomad.broadinstitute.org/, accessed on 5 April 2023) [30], 1000 Genomes Project (http://www.1000genomes.org/, accessed on 5 April 2023) [31], ClinVar (https://www.ncbi.nlm.nih.gov/clinvar/, accessed on 20 April 2023) [32], and Human Gene Mutation Database (HGMD), (http://www.hgmd.cf.ac.uk/ac/index.php, accessed on 5 April 2023) [33] were inquired for the presence of the *SLC22A12* variant identified. Multiple sequence analysis was carried out using Clustal Omega (1.2.4) (Available on: https://www.ebi.ac.uk/Tools/msa/clustalo/, accessed on 10 May 2023) [34]. In addition, we used another bioinformatics tool, ConSurf, to estimate the evolutionary conservation of the altered amino acid positions in the protein (https://consurf.tau.ac.il/) (Accessed on: 16 August 2023) [35].

Varsome, a bioinformatics tool that evaluates the effect of gene variants on protein structure and function, was used to estimate the pathogenicity of the new *SLC22A12* variant (https://varsome.com/) (Accessed on: 5 September 2022) [36]. Variant pathogenicity is informed employing an automatic classifier that evaluates variants according to the American College of Medical Genetics and Genomics (ACMG) guidelines [37]. Variants are classified as pathogenic, likely pathogenic, benign, likely benign, or uncertain significance.

### 2.3. Protein Structural Modeling (Molecular Modeling)

The structural prediction tool DynaMut was used to analyze the impact of mutations on protein structure and thermodynamic stability (https://biosig.lab.uq.edu.au/dynamut2/) (Accessed on: 13 July 2023) [38]. This tool generates a consensus prediction of variations in Gibbs Free Energy (ΔΔG) and its impact on protein stability, where negative values indicate a destabilizing effect. It also allows for the visualization and comparison of the different interactions formed by the wild-type and the mutated residue. The PDB accession numbers used for URAT1 and GLUT9 were Q96S37and Q9NRM0, respectively. Furthermore, we used the web server HOPE to analyze the structural effects of mutations on URAT1 and GLUT9 (https://www3.cmbi.umcn.nl/hope/) (Accessed on: 4 September 2023) [39].

## 3. Results and Discussion

**Figure 1:**
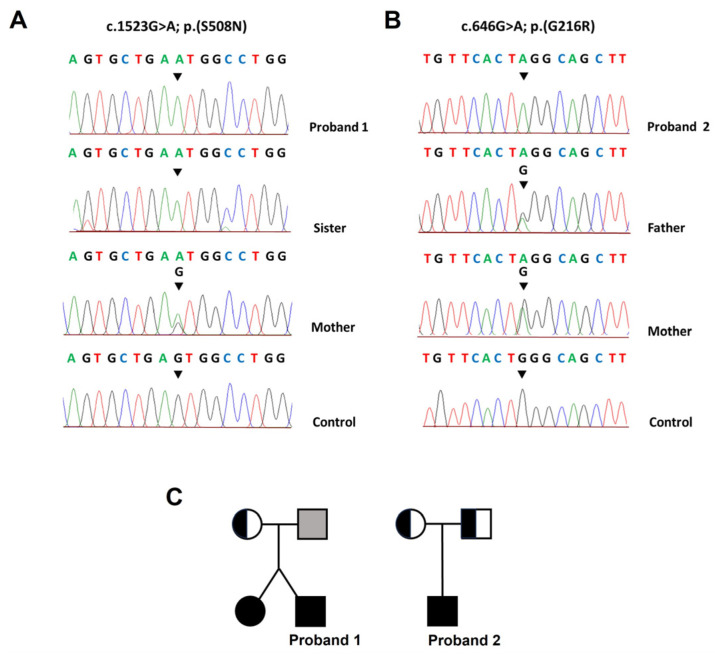
Validation of the variants identified by WES using Sanger sequencing and pedigrees of the two families. Electropherograms show partial DNA sequences of *SLC22A12* (**A**) and *SLC2A9* (**B**) in family members. Arrows indicate the affected nucleotide positions for mutations c.1523G>A; p.(S508N) and c.646G>A; p.(G216R). (**C**) Pedigrees showing the inheritance patterns of the identified variants. Circles and squares represent female and male individuals, respectively; black circles and squares represent affected individuals; black and white circles and squares denote carriers; the grey square represents a family member not available for the genetic analysis.

Reduced UA serum concentrations (<2 mg/dL) and markedly elevated FE UA (>11%) in the two probands led to the suspicion of RHUC. We performed WES analysis after obtaining informed consent and examined variants in genes associated with UA transport. The results showed that proband 1 and his sister were homozygous for missense variant c.1523G>A; p.(S508N) on exon 9 of *SLC22A12* (Ensembl transcript ID: ENST00000377547.1) (Supplementary data, Table S1). In addition, their mother was a heterozygous carrier of the same variant. This mutation substitutes a serine at position 508 for an asparagine in TMD12 of the URAT1 protein. PCR amplification and direct sequencing of exon 9 confirmed these results (Figure 1).

There were no additional rare variants detected by WES analysis in other known UA transport genes (Supplementary data, Table S1). Nor did we detect other rare variants that could explain the phenotype of the two patients (Supplementary data, Table S2). Mutation c.1523G>A; p.(S508N) was not found in gnomAD v2.1.1, the 1000 Genome Project, ClinVar and HGMD databases (accessed on 5 April 2023). Therefore, we believe that this *SLC22A12* variant is novel. We submitted it to ClinVar and it was included with the accession number VCV002501006.1. VarSome analysis classified variant p.(S508N) as of uncertain significance according to the pathogenicity criteria established by ACMG (2 supporting criteria, PM2 and BP4) [40]. Different individual tools rated this variant as damaging or pathogenic (Table 1). In order to gain further insight into the pathogenic effect of the new URAT1 variant, we analyzed its impact on the protein 3-dimensional (3-D) structure.

**Figure 2:**
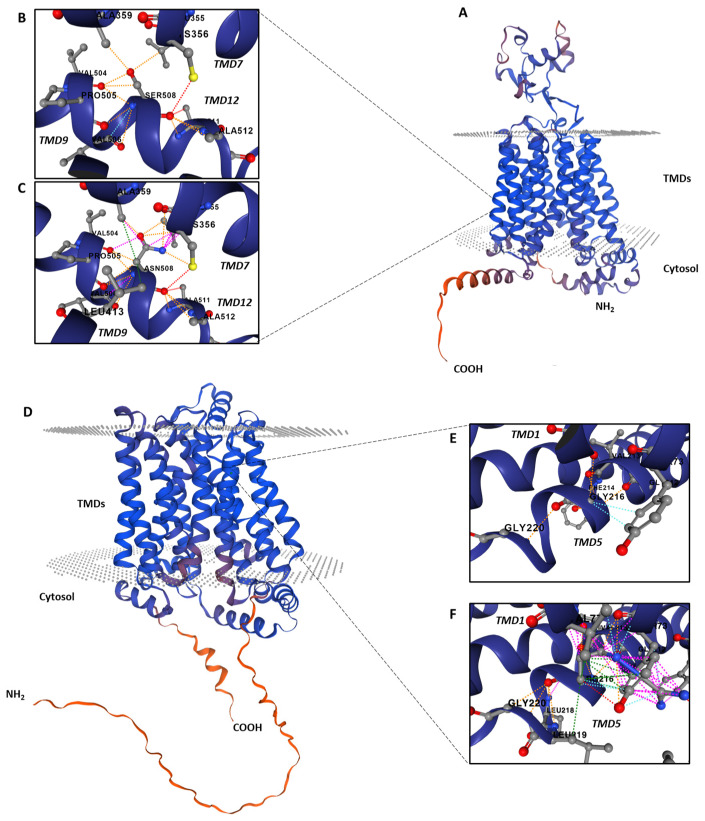
Predicted interactions between amino acid residues in the WT and mutant URAT1 and GLUT9 proteins. (**A**) 3D structure of the WT URAT1 protein. (**B**) Interactions between amino acid residues surrounding the serine 508 in the WT URAT1 protein. (**C**) Interactions between amino acid residues surrounding the mutant residue asparagine. (**D**) 3-D structure of the WT GLUT9 protein. (**E**) Interactions in WT GLUT9 between glycine 216 and its surrounding amino acids. (**F**) New interactions between amino acids produced by the change in glycine 216 to arginine in mutant GLUT9. The WT and mutant residues are represented as sticks together with the neighboring residues that are involved in any kind of interactions. Pink dotted lines indicate new interactions appearing in the mutant protein. Red dotted lines, hydrogen bonds; light blue, van der Waals interactions; orange, polar interactions. TMD, transmembrane domain.

URAT1 belongs to the organic anion transporter (OAT) family [41], a group of proteins characterized by homologous amino acid sequences and similar secondary structures. All of them consist of 12 transmembrane domains (TMDs), with an intracellular loop situated between TMDs 6 and 7 and an extracellular loop located between TMDs 1 and 2. These TMDs are structured into three layers, with each layer serving analogous structural and functional roles [41]. Notably, the outermost TMDs 3, 6, 9, and 12 are critical for maintaining the integrity of transport processes. Wild-type amino acid residue serine 508 is conserved among vertebrate species and other OAT transporters (Figure S1). According to HOPE, this residue, located in TMD12 (Figure 2), and the mutant asparagine differ in size, charge, and hydrophobicity (Figure S2). These modifications also impact the neighboring region, causing significant changes in the interactions between TMD12 and the adjacent TMD7, which include changes in the non-covalent interaction types between TMDs 12 and 7 and the formation of a previously non-existent interaction between the mutant residue (asparagine 508) and leucine 413 located in TMD9 (See 3-D models obtained with DynaMut in Figure 2). As a result, the overall function of the protein is also affected. The absolute value of predicted stability change (ΔΔG_Stability_) for mutation p.(S508N) was −0.37 kcal/mol, which according to DynaMut2 criteria indicates a destabilizing effect. These results together with the low serum UA levels and increased FE UA observed in the proband and his sister suggest that the novel *SLC22A12* mutation, c.1523G>A; p.(S508N), is pathogenic. However, UA transport studies are required to evaluate the functional properties of this novel mutation.

WES analysis in proband 2 identified a homozygous variant, c.646G>A; p.(G216R), in exon 5 of *SLC2A9* (Reference ID: rs561633150. Ensembl transcript ID: ENST00000264784.3). Both parents carried this mutation in heterozygosis. PCR amplification and Sanger sequencing of exon 5 confirmed these results (Figure 1). No additional rare variants were detected by WES analysis in known genes associated with UA transport (Supplementary data, Table S3). Moreover, we did not detect other rare variants that could explain the patient’s phenotype (Supplementary data, Table S4). Variant p.(G216R) occurs in TMD5 of GLUT9 and substitutes a highly conserved glycine residue with a basic charged amino acid [40,41]. VarSome analysis classified this variant as of uncertain significance based on the pathogenicity criteria established by ACMG (1 moderate, PP5, and 2 supporting, PP3 and BP6) [35]. Several individual tools regarded this variant as damaging or pathogenic (Table 1). This variant is only present in the South Asian population (allele frequency 0.00393) and the allele frequency is low in the total population (0.000125) (gnomAD Genomes version 3.1.1, accessed on 5 April 2023).

Variant c.646G>A; p.(G216R), has been previously identified in homozygous or compound heterozygous estate in three RHUC type 2 patients, one of whom is also of Pakistani origin [7,24]. As observed in proband 2 of our study, these three patients had very low UA levels and very high FE UA. Two of these cases presented episodes of acute kidney injury (in two cases induced by intense exercise) [7,42]. Other GLUT9 homozygous or compound heterozygous mutations cause a similar phenotype [6,7,8,42,43,44]. Severe hypouricemia caused by loss-of-function mutations in *SLC2A9* prevent UA absorption through all apical transporters by completely blocking UA efflux [6]. Expression studies with *Xenopus* oocytes have shown that mutation p.(G216R) considerably reduces the transport of UA compared to wild-type GLUT9 as a consequence of a large reduction in expression of the mutant GLUT9 protein [40].

GLUT9 is a member of the GLUT family of glucose transporters [45]. Although it was formerly suggested to transport fructose, subsequent studies reported that GLUT9 was indeed a urate transporter [12]. These proteins have 12 TMDs and intracellular amino and carboxy termini. The wild-type residue glycine 216 is well conserved during evolution and is located in TMD5 of GLUT9 (Figures S1 and S2). The results obtained with DynaMut and HOPE indicate that the mutant arginine residue is bigger than the wild-type glycine residue, which is buried in the core of the protein, and introduces a positive charge possibly leading to protein folding problems (Figure 2 and Figure S2). These changes also impact the neighboring region, causing significant changes in the interactions with different amino acids from TMD5 and TMD1, which include changes in the non-covalent interaction types between amino acids from TMD5 and the formation of a previously nonexistent interaction between the mutant residue (arginine 216) and valine 77 located in TMD1 (Figure 2). As a result, the overall function of the protein is also affected. The absolute value of predicted stability change (ΔΔG_Stability_) for mutation p.(G216R) was—0.65 kcal/mol, which according to DynaMut2 criteria indicates a destabilizing effect.

In our study, the patient with the homozygous *SLC22A12* mutation (proband 1) and her sister had a less severe RHUC (serum UA levels of 0.8 mg/dL, FE UA of 54% and serum UA level of 0.7 mg/dL, FE UA of 40%, respectively) than the patient with the homozygous *SLC2A9* mutation (proband 2). Similar results have been reported in other studies [4,6,8,9,32].

## 4. Conclusions

We described the clinical characteristics of two RHUC probands. Whole exome sequence analysis of the two families revealed a novel missense mutation, p.(S508N), in *SLC22A12*, which affects TMD12 of the URAT1 protein, and a previously reported *SLC2A9* mutation, p.(G216R), that affects TMD5 of GLUT9, both in homozygous estate. The clinical manifestations of the probands were highly consistent with the phenotype of RHUC induced by the homozygous *SLC22A12* and *SLC2A9* variants. Our findings suggest that these loss-of-function mutations cause renal hypouricemia via loss of UA reabsorption and expand the variation spectrum of the *SLC22A12* gene. In addition, this study further emphasizes the importance of WES analysis in clinical settings.

## Figures and Tables

**Table 1:** Bioinformatics predictions of pathogenicity for the two mutations identified in this study ^1^.

Gene	Mutation	Polyphen2 HDIV	Polyphen2 HVAR	SIFT	SIFT4G	PROVEAN	MutPred	Deogen2	EVE	DANN	Mutation Assessor
*SLC22A12*	c.1523G>A p.(S508N)	0.974 (PP)	0.632 (US)	0.002 (US)	0.007 (US)	−2.62 (US)	0.61 (US)	0.57 (US)	-	0.968 (PB)	2.75 (US)
*SLC2A9*	c.646G>A p.(G216R)	0.994 (P)	0.974 (PP)	0.002 (US)	0.005 (US)	−4.38 (PP)	0.908 (P)	0.65 (US)	0.936 (P)	0.999 (PP)	3.2 (PP)

^1^ P: Pathogenic; PP: Probably pathogenic; US: Uncertain significance; PB: Probably benign. Pathogenicity thresholds values: Polyphen2: >0.15; SIFT: <0.05; PROVEAN: <−2.5; MutPred: >0.5; Deogen2: >0.45; EVE: >0.5; DANN: >0.5; Mutation Assessor: >1.95.

## Data Availability

The data sets generated and/or analyzed during the current study are available from the corresponding author upon reasonable request.

## Supplementary Materials

The following supporting information can be downloaded at: https://www.mdpi.com/article/10.3390/genes14091823/s1, Table S1: Variants found in genes related to renal hypouricemia of proband 1 and his sister; Table S2: Rare variants found in WES of proband 1; Table S3: Variants found in genes related to renal hypouricemia of proband 2; Table S4: Rare variants found in WES of proband 2; Figure S1. Evolutionary conservation of amino acid residues affected by the mutations.; Figure S2. Close-up of 3D models of URAT1 (A) and GLUT9 (B) obtained by HOPE.
