# Supplementary material for: New SLC22A12 (URAT1) Variant Associated with Renal Hypouricemia Identified by Whole-Exome Sequencing Analysis and Bioinformatics Predictions
Source: Genes (Basel). 2023 Sep 20;14(9):1823. doi: 10.3390/genes14091823 (PMC10530539; doi:10.3390/genes14091823)
Supplement: Supplementary file 1 [file genes-14-01823-s001.zip › genes-2558334-supplementary.pdf]

Chr.	Position	Ref.	Alt.	Zygosity	Effect	Gene	Ref.	Alt.	SNP ID	Freq.	ClinVar
chr4	9.909.923	G	A	HET	missense_variant	*SLC2A9*	P	L	rs2280205	0,27	Benign
chr4	10.022.981	C	T	HET	missense_variant	*SLC2A9*	G	R	rs2276961	0,41	Benign
chr4	10.027.542	C	T	HET	missense_variant	*SLC2A9*	A	T	rs6820230	0,27	.
chr4	89.013.496	C	T	HET	missense_variant	*ABCG2*	D	N	rs34783571	3,4 x10^{-3}	.
chr6	25.813.150	G	A	HET	missense_variant	*SLC17A1*	T	I	rs1165196	0,72	.
chr6	43.270.151	C	T	HET	splice_region_variant& synonymous_variant	*SLC22A7*	S	S	rs2270860	0,46	.
chr11	**64.368.335**	**G**	**A**	**HOM**	**missense_variant**	***SLC22A12***	**S**	**N**	**rs766798648**	**.**	**.**

Table S1. Variants found in genes related to hypouricemia of proband 1. Variants related to hypouricemia detected by whole exome sequencing in proband 1 after excluding variants in introns and synonymous variants and variants with quality <100. Chr: chromosome, Ref: reference, Var: variant, AA: amino acid, Mut: mutation, Freq: frequency. The genes included in the analysis were: SLC22A12, SLC2A9, SLC22A6, SLC22A8, ABCG2, SLC17A1, SLC17A3, SLC22A7, SLC22A11 and PDZK1. SNP ID rs766798648 represents mutation p.(S508N) (in bold).

Chr.	Position	Ref.	Alt.	Zygosity	Effect	Gene	Ref.	Alt.	SNP ID	Freq.
chr1	53.543.434	C	G	HET	missense_variant	*PODN*	N	K	.	.
chr2	182.399.559	T	A	HET	missense_variant	*ITGA4*	L	Q	rs202038203	$1,9x10^{-4}$
chr3	50.231.314	G	C	HET	splice_region_variant	*GNAT1*	R	P	rs755156826	.
chr3	50.339.759	T	C	HET	missense_variant	*HYAL1*	Y	C	rs1553713151	.
chr4	106.848.523	A	G	HET	missense_variant	*NPNT*	E	G	.	.
chr5	37.341.296	C	T	HET	missense_variant	*NUP155*	R	Q	rs781670166	.
chr5	176.519.361	C	T	HET	missense_variant	*FGFR4*	P	L	rs938855503	.
chr6	105.564.608	A	G	HET	missense_variant	*BVES*	Y	H	.	.
chr7	814.713	G	T	HET	missense_variant	*DNAAF5*	R	L	rs752259246	.
chr7	32.619.853	T	C	HET	missense_variant	*AVL9*	S	P	.	.
**chr11**	**64.368.335**	**G**	**A**	**HOM**	**missense_variant**	***SLC22A12***	**S**	**N**	**rs766798648**	**.**
chr11	125.791.184	C	T	HET	missense_variant	*DDX25*	R	W	rs755709544	.
chr13	60.544.191	A	T	HET	splice_region_variant	*DIAPH3*	I	N	.	.
chr14	89.878.652	C	T	HET	missense_variant	*FOXN3*	D	N	.	.
chr14	92.909.799	C	G	HET	missense_variant	*SLC24A4*	S	C	.	.
chr14	100.793.600	T	A	HET	missense_variant	*SLC25A47*	Y	N	rs759814432	.
chr15	54.614.186	G	T	HET	missense_variant	*UNC13C*	V	F	.	.
chr16	20.477.002	C	T	HET	missense_variant	*ACSM2A*	P	L	rs1295547004	.
chr16	66.547.635	A	G	HET	splice_region_variant	*TK2*	L	P	rs1252881799	.
chr16	70.190.585	G	A	HET	missense_variant	*PDPR*	G	S	rs774550567	.
chr16	85.143.966	G	A	HET	missense_variant	*FAM92B*	R	W	rs753996502	.
chr17	72.306.156	G	A	HET	splice_region_variant	*DNAI2*	V	M	.	.
chr17	72.916.296	C	G	HET	missense_variant	*USH1G*	G	A	rs200197601	$3,9\ x10^{-4}$
chr18	77.659.241	G	A	HET	missense_variant	*KCNG2*	G	R	rs541316487	$1,9\ x10^{-4}$
chr19	10.528.421	C	T	HET	missense_variant	*PDE4A*	P	L	.	.
chr19	17.449.390	C	T	HET	missense_variant	*GTPBP3*	T	I	rs1179310040	.
chr19	37.854.409	A	T	HET	missense_variant	*ZNF875*	H	L	rs746467134	.
chr21	27.071.028	C	G	HET	missense_variant	*JAM2*	A	G	.	.

Table S2. Rare variants found in WES of proband 1. Rare variants detected by whole exome sequencing in proband 1 after excluding variants in introns and UTRs, synonymous variants, variants in databases with frequency >1%, variants with quality <100 and variants with a low probability to be damaging according to SIFT, LRT, MutationTaster, PROVEAN, MutPred and FATHMM. Chr: chromosome, Ref: reference, Var: variant, AA: amino acid, Mut: mutation, Freq: frequency. SNP ID rs766798648 represents mutation p.(S508N) (in bold).

Chr.	Position	Ref.	Alt.	Zygosity	Effect	Gene	Ref.	Var.	SNP ID	Frec.	ClinVar
chr4	9.909.923	G	A	HOM	missense_variant	*SLC2A9*	P	L	rs2280205	0,27	Benign
chr4	9.922.130	C	T	HOM	missense_variant	*SLC2A9*	R	H	rs3733591	0,29	Benign
chr4	**9.982.251**	**C**	**T**	**HOM**	**missense_variant**	***SLC2A9***	**G**	**R**	**rs561633150**	**$9{,}9x10^{-4}$**	**Uncertain_significance**
chr4	10.027.509	GA	G	HOM	upstream_gene_variant	*SLC2A9*	-	-	rs61256984	0,27	.
chr4	10.027.542	C	T	HOM	missense_variant	*SLC2A9*	A	T	rs6820230	0,27	.
chr4	10.027.643	A	G	HOM	upstream_gene_variant	*SLC2A9*	-	-	rs6449237	0,27	.
chr4	10.027.744	G	A	HOM	upstream_gene_variant	*SLC2A9*	-	-	rs6449238	0,27	.
chr4	89.080.270	CAAACACT	C	HET	upstream_gene_variant	*ABCG2*	-	-	rs57327643	0,03	.
chr6	25.813.150	G	A	HOM	missense_variant	*SLC17A1*	T	I	rs1165196	0,72	.
chr6	25.862.466	C	T	HET	missense_variant	*SLC17A3*	A	T	rs1165165	0,22	.
chr6	43.270.151	C	T	HOM	splice_region_variant& synonymous_variant	*SLC22A7*	S	S	rs2270860	0,46	.

Table S3. Variants found in genes related to renal ehypouricemia of proband 2. Variants related to hypouricemia detected by whole exome sequencing in proband 2 after excluding variants in introns and synonymous variants and variants with quality <100. Chr: chromosome, Ref: reference, Var: variant, AA: amino acid, Mut: mutation, Freq: frequency. The genes included in the analysis were: *SLC22A12, SLC2A9, SLC22A6, SLC22A8, ABCG2, SLC17A1, SLC17A3, SLC22A7, SLC22A11* and *PDZK1*. SNP ID rs561633150 represents mutation p.(G216R) (in bold).

Chr.	Position	Ref.	Alt.	Zygosity	Effect	Gene	Ref.	Alt.	SNP ID	Freq.
chr1	45.796.892	C	A	HET	stop_gained	*MUTYH*	E	X	rs121908381	0,002
chr1	54.605.318	T	TG	HOM	frameshift_variant	*CDCP2*	-	-	rs3841798	.
chr1	144.866.643	G	A	HET	missense_variant	*PDE4DIP*	R	C	rs1620560	.
chr4	87.313	G	GA	HOM	frameshift_variant	*ZNF595*	-	-	rs60154095	.
**chr4**	**9.982.251**	**C**	**T**	**HOM**	**missense_variant**	***SLC2A9***	**G**	**R**	**rs561633150**	**$9,9x10^{-4}$**
chr4	140.811.083	GC	G	HOM	frameshift_variant	*MAML3*	-	-	rs373804063	.
chr5	175.811.094	C	CGT	HOM	frameshift_variant	*NOP16*	-	-	rs56989856	.
chr8	144.947.015	G	T	HET	missense_variant	*EPPK1*	A	D	rs782720515	.
chr10	127.350.414	C	T	HET	splice_donor_variant&intron_variant	*TEX36*	-	-	rs1340966227	.
chr11	1.213.416	G	A	HET	missense_variant	*MUC5AC*	R	Q	rs78511643	.
chr11	56.143.255	T	TGA	HET	frameshift_variant	*OR8U8*	-	-	rs754716745	.
chr14	20.666.175	C	CA	HET	frameshift_variant	*OR11G2*	-	-	rs55781225	.
chr16	31.470.799	TG	T	HET	frameshift_variant	*ARMC5*	-	-	.	.
chr16	56.904.646	A	C	HET	missense_variant&splice_region_variant	*SLC12A3*	K	Q	rs200086762	.
chr16	89.266.189	C	T	HET	missense_variant	*SLC22A31*	R	H	rs536851687	.
chr17	21.204.210	C	T	HET	stop_gained	*MAP2K3*	Q	X	rs55796947	.
chr17	21.319.087	G	A	HET	missense_variant	*KCNJ12*	G	S	rs75029097	$1,9x10^{-4}$
chr17	73.729.656	G	A	HET	missense_variant	*ITGB4*	G	S	rs746401769	.
chr19	44.933.010	ACT	A	HET	frameshift_variant	*ZNF229*	-	-	rs772945873	.
chr19	45.659.155	G	C	HET	missense_variant	*NKPD1*	D	E	rs144764378	0,002
chr22	24.041.250	T	C	HET	missense_variant	*RGL4*	W	R	rs559617660	0,001
chr22	42.457.056	C	T	HET	missense_variant	*NAGA*	E	K	rs121434529	$3,9x10^{-4}$
chr22	43.926.845	C	T	HET	splice_acceptor_variant&intron_variant	*EFCAB6*	-	-	rs376668505	0,001

Table S4. Rare variants found in WES of proband 2. Rare variants detected by whole exome sequencing in proband 2 after excluding variants in introns and UTRs, synonymous variants, variants in databases with frequency >1%, variants with quality <100 and variants with a low probability to be damaging (score <0,80 in Polyphen). Chr: chromosome, Ref: reference, Var: variant, AA: amino acid, Mut: mutation, Freq: frequency. SNP ID rs561633150 represents mutation p.(G216R) (in bold).

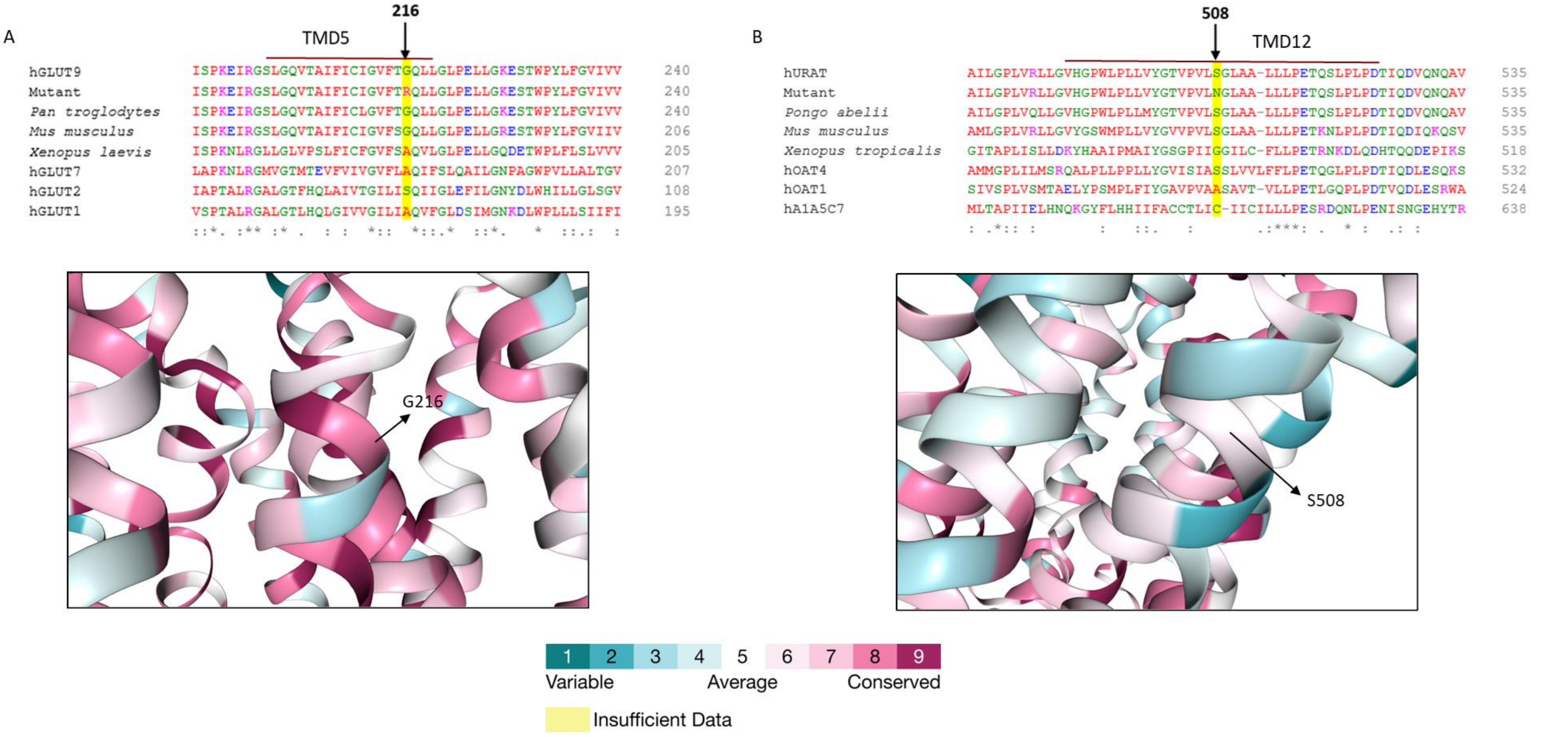

Figure S1. Evolutionary conservation of amino acid residues affected by the mutations. Panels A and B show multiple alignments of GLUT9 and URAT1 regions affected, respectively, with a subset of vertebrate orthologs and other transporters, obtained with Clustal Omega. The arrows mark the amino acid residues affected by the respective mutations and the red line indicates the TMDs in which they are found. Panel C shows the model obtained from Consurf, in which the conservation of amino acids is observed according to a color scale. The arrow marks the mutated amino acids.

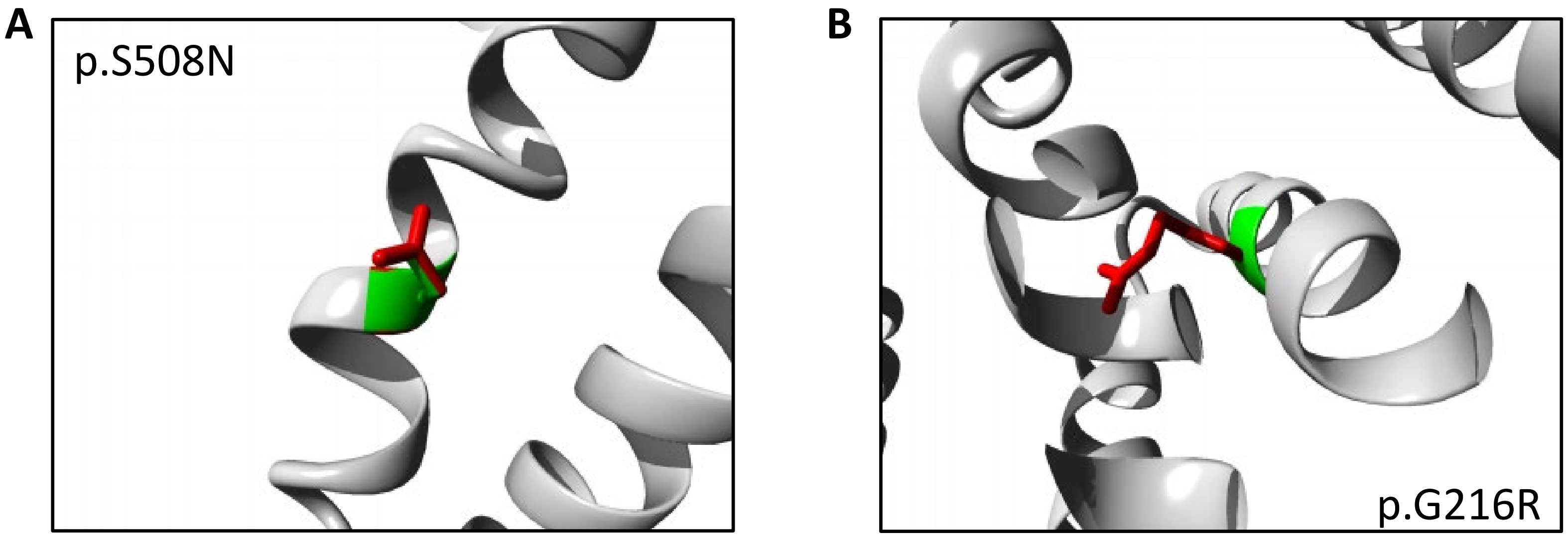

Figure S2. Close-up of 3D models of URAT1 (A) and GLUT9 (B) obtained by HOPE. The protein is coloured grey, the side chains of both the wild-type and the mutant residues (p.S508N and p.G216R) are shown and coloured green and red, respectively.
